# Outcomes of active surveillance in low-risk prostate cancer: A retrospective cohort study

**DOI:** 10.1097/MD.0000000000045691

**Published:** 2025-10-24

**Authors:** Dursun Baba, Melih Balçin, Yusuf Şenoğlu, Ahmet Yildirim Balik, Ekrem Başaran, Arda Taşkin Taşkiran, Mehmet Ali Özel, Muhammet Ali Kayikçi

**Affiliations:** a Department of Urology, Duzce University, Faculty of Medicine, Duzce, Türkiye; b Department of Urology, Marmara University Faculty of Medicine, İstanbul, Türkiye; c Department of Radiology, Duzce University Faculty of Medicine, Duzce, Türkiye.

**Keywords:** active surveillance, multiparametric magnetic resonance imaging, prostate biopsy, prostate cancer

## Abstract

Active surveillance (AS) is an established strategy for managing low-risk prostate cancer (LRPCa), aiming to reduce overtreatment while maintaining oncological safety. This retrospective cohort study included 102 patients diagnosed with LRPCa between 2015 and 2025, managed with serial prostate-specific antigen (PSA) testing, digital rectal examination, multiparametric MRI (mpMRI), and confirmatory biopsies. Transition criteria included Gleason score upgrading, PSA progression, lesion progression on mpMRI, or patient preference. The median follow-up was 16 months (range: 6–123), while the estimated median surveillance duration was 36 months (95% confidence interval: 30–42). Retention rates at 2 and 5 years were 72% and 50%, respectively. A total of 35.3% (36/102) of patients discontinued AS, most frequently due to patient preference (61.1%), followed by PSA progression (25.0%) and histopathological upgrading (13.9%). Only 25.5% (26/102) of patients underwent confirmatory biopsy during follow-up, reflecting suboptimal adherence that may have contributed to an underestimation of true pathological progression. Multivariate analysis revealed that higher final PSA levels (odds ratio [OR]: 1.559, *P* = .001), abnormal digital rectal examination findings (OR: 11.079, *P* < .001), and Prostate Imaging-Reporting and Data System 4 to 5 lesions on mpMRI (OR: 5.482, *P* < .001) were independently associated with transition to definitive treatment. These findings suggest that AS remains a feasible and effective management strategy for LRPCa, though confirmatory biopsies and structured psychological support are essential to optimize adherence and prevent overtreatment. Future studies should investigate the integration of biomarkers and AI-assisted imaging in refining AS protocols.

## 1. Introduction

Prostate cancer is one of the most commonly diagnosed malignancies in men worldwide and ranks 2nd after lung cancer in terms of cancer-related mortality.^[1,2]^ With advances in early detection methods and the widespread use of prostate-specific antigen (PSA) screening, the diagnosis of clinically insignificant, low-risk prostate cancer (LRPCa) has become increasingly common.^[3,4]^ LRPCa is often an indolent disease, and many patients can live with the condition without experiencing significant clinical progression throughout their lifetime.^[5,6]^

Traditionally, localized prostate cancer has been managed with curative treatment options such as radical prostatectomy (RP) or radiotherapy (RT). However, these aggressive interventions are associated with significant adverse effects, including erectile dysfunction, urinary incontinence, and bowel dysfunction, which can substantially impact the quality of life.^[7,8]^ Recent studies have demonstrated that active surveillance (AS) is a viable strategy for low-risk patients, effectively delaying or avoiding unnecessary treatment while maintaining oncological safety.^[9,10]^

Active surveillance involves the careful monitoring of patients through serial PSA measurements, digital rectal examinations (DRE), and periodic prostate biopsies, with definitive treatment initiated if disease progression is detected.^[11]^ Both the European Association of Urology and the National Comprehensive Cancer Network recommend AS as a primary approach for LRPCa patients. However, there is still no clear consensus regarding the optimal selection criteria for AS candidates and the most reliable predictors of disease progression.^[12]^

In recent years, multiparametric magnetic resonance imaging (mpMRI) and fusion biopsy have gained increasing importance in AS, yet their long-term impact on disease management remains unclear.^[13]^ The literature presents ongoing debates regarding the optimal duration of surveillance, the criteria for transitioning to definitive treatment, and the most significant predictors of progression.^[14,15]^

This study aims to evaluate the clinical and oncological outcomes of 102 patients undergoing AS at a single center. We seek to assess the effectiveness of AS, identify factors associated with disease progression, and determine which patient groups are most suitable for long-term surveillance. Additionally, we will analyze the relationship between biopsy findings, PSA kinetics, and mpMRI results, as well as the rates and reasons for transitioning to curative treatment, contributing valuable insights to the existing literature.

## 2. Materials and methods

### 2.1. Study design and ethical approval

This study was designed as a single-center, retrospective cohort study, including patients diagnosed with LRPCa who were enrolled in the AS protocol between 2015 and 2025 at the Department of Urology, Düzce University. Ethical approval was obtained from the Düzce University Clinical Research Ethics Committee (Ethics Committee Decission No: 2025/53, February 24, 2025), and the study was conducted in accordance with the Declaration of Helsinki. Written informed consent was obtained from all patients included in the study.

### 2.2. Patient selection and inclusion criteria

The inclusion criteria were determined based on the guidelines of the European Association of Urology and the National Comprehensive Cancer Network. Patients meeting the following criteria were included in the study: PSA level < 10 ng/mL, Gleason score ≤ 6 (3 + 3), clinical stage ≤ T2A, and those who underwent mpMRI.^[16]^

Exclusion criteria included patients with PSA > 10 ng/mL, Gleason score ≥ 7 (4 + 3) or higher-grade tumors, clinical stage ≥ T2B, prior treatment with RT or RP, and those with incomplete follow-up data.^[16]^

### 2.3. Follow-up protocol

At the initial assessment, mpMRI was performed on all patients, and for those with Prostate Imaging-Reporting and Data System (PI-RADS) ≥ 3, additional fusion biopsy was conducted. Patients were monitored at 3-month intervals with PSA measurements and DRE.

From the 1st year onward, annual prostate biopsies were recommended, with biopsy protocols structured as follows: patients with negative mpMRI findings underwent a systematic 12-core biopsy, while those with PI-RADS ≥ 3 underwent mpMRI-fusion biopsy in addition to the standard 12-core biopsy.

Criteria for discontinuation of AS and transition to definitive treatment included Gleason score progression (ISUP Grade 2 or higher), a significant increase in PSA velocity or a PSA doubling time of less than 3 years, newly detected lesions or significant progression of existing lesions on mpMRI, and patient preference for curative treatment. Patients who discontinued AS underwent either RP or RT as definitive treatment options.^[14,17]^

### 2.4. Biopsy protocol

All patients underwent a pre-biopsy urine culture, and those with positive results were treated with appropriate antibiotics before undergoing the procedure. Patients undergoing anticoagulant therapy were referred to the appropriate department for consultation. When deemed clinically necessary, their regimen was transitioned to a short-acting anticoagulant protocol prior to the biopsy.

A sterile lubricating gel (Cathajell 12.5 g) was rectally applied to provide local analgesia, followed by the administration of 10 cc of local anesthesia (Citanest 2%) bilaterally between the prostate and seminal vesicles using a 20G, 25 cm aspiration needle under transrectal ultrasound guidance. Patients subsequently underwent either a systematic 12-core biopsy or a mpMRI-fusion biopsy. The collected biopsy specimens were fixed in formalin and submitted for histopathological analysis. Following the procedure, all patients were monitored for 3 hours and discharged with ciprofloxacin 500 mg oral antibiotic prophylaxis prescribed twice daily after the 1st spontaneous voiding.^[18]^

### 2.5. Statistical analysis and sample size calculation

Data were analyzed using IBM SPSS version 20 (Chicago). Continuous variables were expressed as mean ± standard deviation, median (minimum–maximum), and categorical variables were presented as frequency and percentage (%). Comparisons between groups were performed using independent *t* test for normally distributed variables and Mann–Whitney *U* test for non-normally distributed variables. Nominal variables were analyzed using Chi-square test or Fisher exact test.

To evaluate PSA changes over time, the difference between initial and final PSA values was analyzed using the Wilcoxon test. The relationships between PSA kinetics, tumor burden, and lesion size were assessed using Spearman correlation coefficient. Predictors of transition to definitive treatment were evaluated using multivariate logistic regression analysis, with results reported as adjusted odds ratios (OR) and 95% confidence intervals (CI). Kaplan–Meier survival analysis was used to estimate the duration of AS, and survival differences between independent groups were compared using the Log-Rank test. A *P*-value < .05 was considered statistically significant.

Sample size calculation was performed using G*Power software to determine the minimum number of patients required for statistical significance. Based on a power (1-β) of 80%, an effect size of 0.5, and a significance level (α) of 0.05, the minimum required sample size was calculated as 54 patients.^[19]^ However, to increase the robustness of the findings and account for potential dropouts, a total of 102 patients were included in the study.

## 3. Results

According to Table 1, a total of 102 patients were included in the study. The median age was 68 years (range: 46–80), and the median follow-up duration was 16 months (range: 6–123 months). The mean pre-biopsy PSA level was 5.49 ± 1.75 ng/mL, while the mean PSA level at the final follow-up was 6.65 ± 2.30 ng/mL. Abnormal findings on DRE were detected in 37.3% of patients. mpMRI findings showed 50% of patients had PI-RADS 2 lesions, 17.6% had PI-RADS 3, 24.5% had PI-RADS 4, and 7.8% had PI-RADS 5 lesions.

**Table 1:** Baseline characteristics of the patients.

Variable	n = 102	Median (min–max)	Mean ± SD
Age (yr)	102	68 (46–80)	67.15 ± 6.28
Follow-up duration (mo)	102	16 (6–123)	26.75 ± 23.53
Pre-biopsy PSA (ng/mL)	102	-	5.49 ± 1.75
Final PSA (ng/mL)	102	-	6.65 ± 2.30
Abnormal DRE, n (%)	102	38 (37.3%)	–
MRI lesion, n (%)	102		
PI-RADS 2	51 (50.0%)		
PI-RADS 3	18 (17.6%)		
PI-RADS 4	25 (24.5%)		
PI-RADS 5	8 (7.8%)		
Prostate volume (cc)	102	45 (11-107)	47.75 ± 21.40
Prostate density	102	0.11 (0.01-0.51)	0.14 ± 0.09
Lesion size (mm)	45	68.8 (12.0–544.0)	112.62 ± 118.77
Positive biopsy foci	102	2 (1–10)	2.48 ± 1.89
Tumor involvement (%)	102	10 (1–50)	15.22 ± 12.85

DRE = digital rectal examination, Max = maximum, Min = minimum, MRI = magnetic resonance imaging, PI-RADS = Prostate Imaging-Reporting and Data System, PSA = prostate-specific antigen, SD = standard deviation.

**Figure 1.**
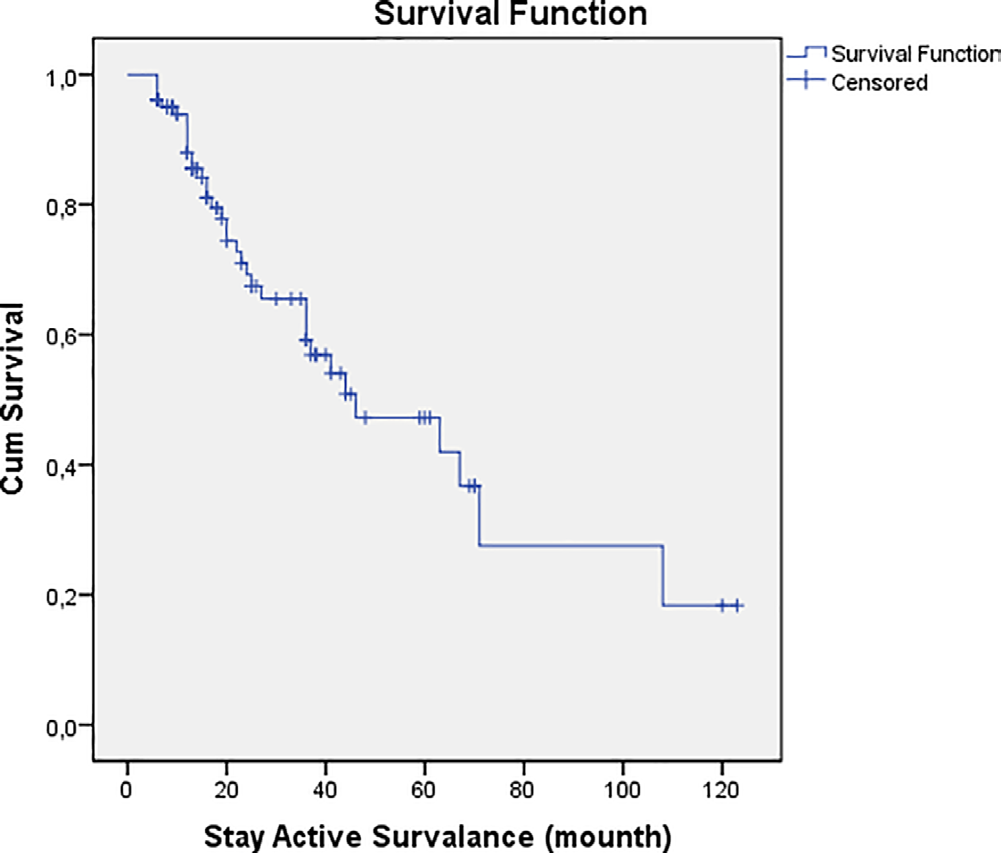
Kaplan–Meier curve for overall active surveillance retention rates.

Figure 1 demonstrates the overall surveillance retention rates over time, showing the percentage of patients who remained on AS at different follow-up intervals.

According to Table 2, PSA levels showed an increasing trend over time. The mean PSA level at the first follow-up was 5.72 ± 2.86 ng/mL, while at the final follow-up it was 6.65 ± 2.30 ng/mL. A significant increase in PSA levels was observed over time (*P* < .001, Wilcoxon test).

**Table 2:** PSA levels during follow-up.

Follow-up time	n	Mean ± SD	Median (min–max)
Initial PSA	102	5.72 ± 2.86	5.2 (1.1–23.7)
3rd Month PSA	102	5.63 ± 2.23	5.5 (0.5–11.8)
6th Month PSA	100	6.19 ± 2.29	6.1 (0.7–12.9)
12th Month PSA	49	5.66 ± 2.74	5.6 (0.5–12.1)
Final PSA	102	6.65 ± 2.30	6.3 (0.8–13.2)

Max = maximum, Min = minimum, PSA = prostate-specific antigen, SD = standard deviation.

As shown in Table 3, 35.3% of patients (n = 36) transitioned from AS to definitive treatment. The most common reason for definitive treatment was patient preference (61.1%), followed by PSA progression (25.0%) and histopathological progression on confirmatory biopsy (13.9%). Among those who received definitive treatment, 77.8% underwent RP, while 22.2% received RT.

**Table 3:** Reasons for treatment transition and treatment modalities.

Variable	n (%)
Patients remaining in active surveillance	66 (64.7%)
Patients undergoing definitive treatment	36 (35.3%)
Reasons for definitive treatment transition:	
Patient preference	22 (61.1%)
PSA progression	9 (25.0%)
Gleason score progression	5 (13.9%)
Treatment type:	
Radical prostatectomy (RP)	28 (77.8%)
Radiotherapy (RT)	8 (22.2%)

PSA = prostate-specific antigen.

According to Table 4, no significant difference was found in age between patients who remained in surveillance and those who transitioned to treatment (*P* = .651). However, PSA levels at the final follow-up were significantly higher in patients who received treatment (7.91 ± 2.77 vs 5.96 ± 1.65, *P* < .001). An abnormal DRE was also significantly associated with transitioning to treatment (*P* < .001).

**Table 4:** Comparison between surveillance and treatment groups.

Variable	Surveillance (n = 66)	Treatment (n = 36)	*P*-value
Age (yr)	69.17 ± 5.95	67.11 ± 6.92	.651
Pre-biopsy PSA	5.30 ± 1.53	5.85 ± 2.06	.166
Final PSA	5.96 ± 1.65	7.91 ± 2.77	<.001
Positive biopsy foci	2.15 ± 1.66	3.08 ± 2.14	.013
Abnormal DRE (%)	18.2%	72.2%	<.001

DRE = digital rectal examination, PSA = prostate-specific antigen.

Multivariate logistic regression analysis identified final PSA levels (*P* = .001, OR: 1.559, 95% CI: 1.203–2.020) and abnormal DRE findings (*P* < .001, OR: 11.079, 95% CI: 3.765–32.601) as significant predictors of transition to definitive treatment. Patients with higher PSA levels and abnormal DRE findings had a significantly higher likelihood of requiring curative treatment.

Additionally, Kaplan–Meier survival analysis was performed to assess the duration of AS. According to Table 5, the estimated median surveillance duration was 36 months (95% CI: 30–42 months). The 2-year and 5-year surveillance retention rates were 72.0% and 50.0%, respectively. Patients with PSA levels above 7 ng/mL and PI-RADS 4–5 lesions had a significantly lower probability of remaining on AS (Log-Rank *P* < .001).

**Table 5:** Multivariate logistic regression analysis of risk factors for transition to treatment and Kaplan–Meier survival outcomes.

Variable	Adjusted OR	95% CI	*P*-value
Final PSA	1.559	1.203–2.020	.001
Abnormal DRE	11.079	3.765–32.601	<.001
PI-RADS 4–5	5.482	2.115–14.207	<.001
PSA > 7 ng/mL	4.312	1.987–9.356	.002
Surveillance duration (median)	36 mo	(95% CI: 30–42)	–
2-year retention rate	72.0%	–	–
5-year retention rate	50.0%	–	–
Log-Rank *P*-value	–	–	<.001

CI = confidence interval, DRE = digital rectal examination, OR = odds ratio, PI-RADS = Prostate Imaging-Reporting and Data System, PSA = prostate-specific antigen.

**Figure 2.**
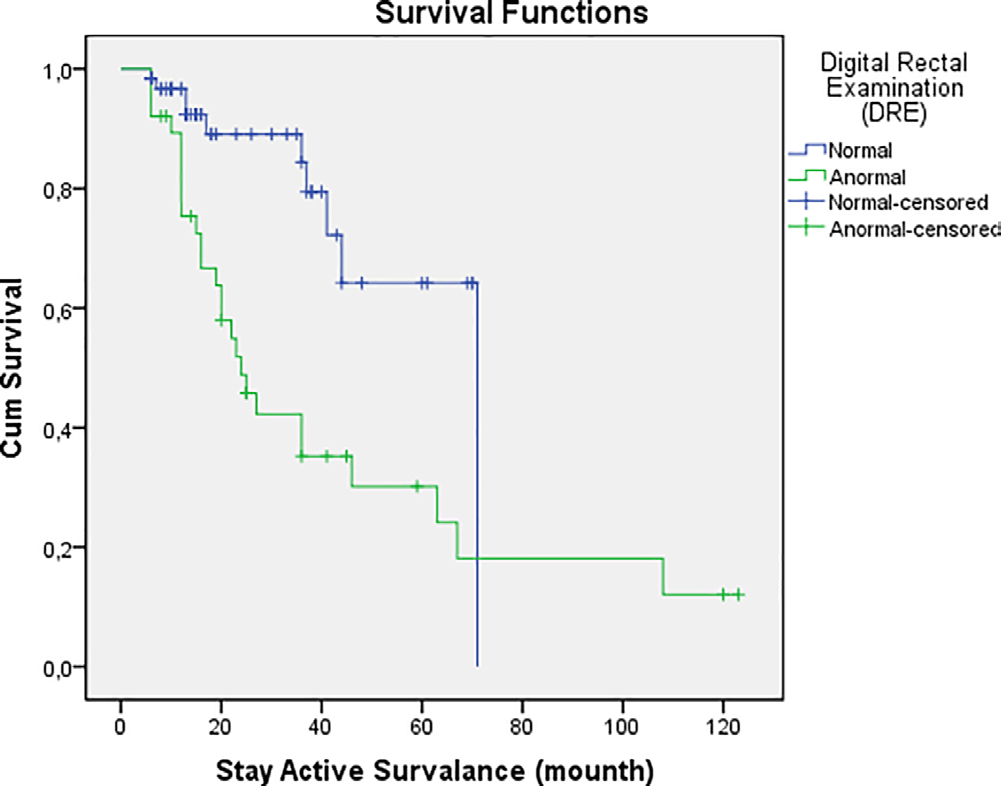
Kaplan–Meier curve for active surveillance retention based on digital rectal examination findings.

Figure 1 illustrates the general surveillance retention rates over time, while Figure 2 specifically demonstrates the impact of abnormal DRE findings on surveillance duration.

## 4. Discussion

This study is a single-center retrospective cohort analysis evaluating the clinical and oncological outcomes of patients undergoing AS for LRPCa. The findings indicate that 64.7% of patients remained on AS, while 35.3% transitioned to definitive treatment. The primary reasons for treatment conversion were identified as patient preference (61.1%), PSA progression (25%), and Gleason score upgrading (13.9%). Additionally, final PSA levels, abnormal DRE findings, and PI-RADS 4 to 5 lesions on mpMRI were found to be significant predictors of treatment transition, emphasizing the importance of a personalized and risk-adapted approach to AS.

Active surveillance has become an increasingly accepted strategy in the management of LRPCa, as endorsed by the European Association of Urology and the National Comprehensive Cancer Network.^[20,21]^ Large-scale studies have demonstrated that 60% to 80% of patients remain on AS for extended periods,^[22,23]^ which is consistent with our 64.7% retention rate. Similarly, Ongun et al^[24]^ reported that 30% to 40% of AS patients eventually transition to curative treatment, closely aligning with our study’s 35.3% treatment transition rate. Although PSA progression and Gleason score upgrading are widely recognized as the most common reasons for treatment transition,^[25,26]^ our study uniquely highlights that patient preference played a dominant role (61.1%). This finding suggests that psychosocial factors such as anxiety, fear of disease progression, cultural perceptions, and physician–patient communication may significantly influence decision-making during AS. Shelton et al^[27]^ similarly found that up to 40% of AS patients opt for treatment due to anxiety rather than clinical progression, underscoring the need for structured psychological support and counseling programs to reduce overtreatment driven by patient anxiety.

One of the most debated aspects of AS is the role of mpMRI and its influence on biopsy frequency and treatment decisions. In our study, PI-RADS 4 to 5 lesions were significantly associated with treatment transition (*P* < .001), supporting the argument that mpMRI is a valuable tool for risk stratification in AS.^[28]^ Multiple studies have suggested that patients with PI-RADS 4 to 5 lesions have a higher likelihood of harboring clinically significant prostate cancer, necessitating closer follow-up and timely biopsy interventions.^[14,29]^ However, the over-reliance on mpMRI findings without confirmatory biopsy may lead to misclassification and delayed treatment. In our cohort, only 26 patients (25.5%) underwent confirmatory biopsy during follow-up, indicating suboptimal adherence to the annual biopsy protocol. This low compliance may have contributed to an underestimation of pathological progression, consistent with prior reports that favorable mpMRI findings reduce patient willingness to undergo scheduled biopsies.^[30]^ Hettiarachchi et al^[13]^ demonstrated that serial mpMRI alone is insufficient, reinforcing that confirmatory biopsies remain important to avoid missing clinically significant tumors. Our findings support this perspective, indicating that mpMRI should complement, but not replace, routine biopsy protocols.

The Kaplan–Meier survival analysis in our study revealed that the median AS duration was 36 months (95% CI: 30–42 months), with 2- and 5-year surveillance retention rates of 72% and 50%, respectively. These figures are comparable to long-term AS cohort studies,^[15,16]^ highlighting that most patients can safely remain on AS for extended periods without oncological compromise.^[31,32]^ However, our study also emphasizes the importance of PSA kinetics in guiding surveillance decisions, as PSA levels above 7 ng/mL were significantly associated with treatment transition (*P* < .001). This supports previous findings suggesting that PSA kinetics, including PSA doubling time and velocity, are crucial for determining biopsy timing and treatment conversion risk.^[33,34]^

An important finding of our study is that abnormal DRE findings independently predicted treatment transition (*P* < .001, OR: 11.079), aligning with previous research demonstrating that suspicious nodules on physical examination often indicate disease progression, even in the absence of biopsy-confirmed changes.^[35]^ This underscores the continued relevance of DRE in AS protocols, despite the increasing reliance on advanced imaging modalities.

While our study provides valuable clinical insights, it also has several limitations. As a single-center retrospective study, its generalizability is inherently restricted. Additionally, the low number of confirmatory biopsies may have led to an underestimation of true progression rates, as some patients refused repeat biopsies after receiving favorable MRI results. This reflects an ongoing shift in AS management, where patients and clinicians increasingly rely on imaging over tissue sampling, potentially affecting treatment decisions. Another significant limitation is that a large proportion of patients (61.1%) transitioned to treatment electively, highlighting the psychological burden of AS. Moreover, although PSA kinetics such as doubling time and velocity are recognized as important predictors of disease progression, our analysis primarily focused on static PSA values. This may have limited the ability to fully capture dynamic changes in PSA over time. These findings suggest that future AS protocols should integrate structured psychological support programs to enhance patient adherence, as well as more comprehensive use of PSA kinetics and biopsy data to minimize bias.

Despite these limitations, our study has several strengths. The long follow-up period (2015–2025) provides a robust evaluation of AS outcomes, and the integration of mpMRI and fusion biopsy data offers critical insights into modern surveillance approaches. Furthermore, the study thoroughly examines PSA kinetics, Gleason score progression, and imaging findings as predictive markers for treatment transition. These results highlight the need for a tailored, risk-adapted approach to AS, ensuring that patients are neither overtreated nor undertreated based on a single diagnostic modality.

The findings of our study have several important clinical implications. First, PSA kinetics remain a cornerstone of AS decision-making, and a PSA threshold of > 7 ng/mL may necessitate closer follow-up and confirmatory biopsies. Second, abnormal DRE findings should not be overlooked, as they were identified as a strong predictor of progression, independent of imaging results. Third, although mpMRI is a valuable risk stratification tool, final decisions should be made with confirmatory biopsies rather than mpMRI evaluation alone in patients with PI-RADS 3 to 5 lesions. Finally, the high rate of treatment transitions due to patient preference highlights the necessity of structured psychological counseling in AS programs to mitigate anxiety-related overtreatment.

Future research should focus on optimizing AS protocols by incorporating novel biomarkers (e.g., genomic classifiers, liquid biopsy techniques) to improve risk stratification and disease progression prediction. Additionally, the integration of artificial intelligence-driven imaging analysis may enhance mpMRI interpretation accuracy and reduce interobserver variability. Standardizing biopsy frequency and assessing the long-term oncological safety of less invasive AS strategies in multi-center prospective studies will be crucial for refining current surveillance approaches.

## 5. Conclusions

In conclusion, AS appears to be a feasible and effective management strategy for LRPCa, providing a reasonable balance between oncological safety and quality-of-life preservation. Nonetheless, careful integration of PSA kinetics, mpMRI findings, systematic biopsies, and psychological counseling may help optimize patient selection and support long-term adherence to surveillance protocols.

## Acknowledgments

The authors would like to thank the clinical staff involved in patient management during the study period.

## Author contributions

**Conceptualization:** Dursun Baba, Melih Balçin.

**Data curation:** Dursun Baba, Melih Balçin, Yusuf Şenoğlu, Mehmet Ali Özel.

**Formal analysis:** Dursun Baba, Melih Balçin.

**Funding acquisition:** Dursun Baba.

**Investigation:** Dursun Baba, Melih Balçin.

**Methodology:** Dursun Baba, Melih Balçin.

**Project administration:** Dursun Baba, Melih Balçin.

**Resources:** Dursun Baba, Melih Balçin, Yusuf Şenoğlu, Ahmet Yildirim Balik, Ekrem Başaran, Arda Taşkin Taşkiran, Mehmet Ali Özel, Muhammet Ali Kayikçi.

**Software:** Dursun Baba, Melih Balçin.

**Supervision:** Dursun Baba, Melih Balçin.

**Validation:** Dursun Baba, Melih Balçin.

**Visualization:** Dursun Baba, Melih Balçin.

**Writing – original draft:** Dursun Baba, Melih Balçin, Yusuf Şenoğlu, Mehmet Ali Özel, Muhammet Ali Kayikçi.

**Writing – review & editing:** Dursun Baba, Melih Balçin, Ahmet Yildirim Balik, Ekrem Başaran, Arda Taşkin Taşkiran.
